# Interplay between innate immunity and the viral oncoproteins Tax and HBZ in the pathogenesis and therapeutic response of HTLV-1 associated adult T cell leukemia

**DOI:** 10.3389/fimmu.2022.957535

**Published:** 2022-07-22

**Authors:** Hiba El Hajj, Ali Bazarbachi

**Affiliations:** ^1^ Department of Experimental Pathology, Immunology and Microbiology, Faculty of Medicine, American University of Beirut, Beirut, Lebanon; ^2^ Department of Internal Medicine, Faculty of Medicine, American University of Beirut, Beirut, Lebanon; ^3^ Department of Anatomy, Cell Biology and Physiological Sciences, Faculty of Medicine, American University of Beirut, Beirut, Lebanon

**Keywords:** HTLV-1/HTLV-1, ATL/ATLL, Tax, HBZ, innate immunity, interleukin-10 (IL-10), targeted therapy/therapies

## Abstract

The Human T-cell Leukemia virus type 1 (HTLV-1) causes an array of pathologies, the most aggressive of which is adult T-cell leukemia (ATL), a fatal blood malignancy with dismal prognosis. The progression of these diseases is partly ascribed to the failure of the immune system in controlling the spread of virally infected cells. HTLV-1 infected subjects, whether asymptomatic carriers or symptomatic patients are prone to opportunistic infections. An increasing body of literature emphasizes the interplay between HTLV-1, its associated pathologies, and the pivotal role of the host innate and adoptive immune system, in shaping the progression of HTLV-1 associated diseases and their response to therapy. In this review, we will describe the modalities adopted by the malignant ATL cells to subvert the host innate immune response with emphasis on the role of the two viral oncoproteins Tax and HBZ in this process. We will also provide a comprehensive overview on the function of innate immunity in the therapeutic response to chemotherapy, anti-viral or targeted therapies in the pre-clinical and clinical settings.

## 1 Human T-cell lymphotropic virus type I-associated diseases: A brief overview with emphasis on adult T cell leukemia

The Human T-cell leukemia virus type 1 (HTLV-1) is the first oncogenic retrovirus associated with a human disease (1). HTLV-1 endemicity spans several continents, including Central and Latin America, the Caribbean islands, Southern Japan, Intertropical Africa, Romania, North-East Iran in the Middle East, Melanesia and Central Australia (2–6). Around 20 million people are infected worldwide, with only 5–10% who develop diseases, depending on their ethnic origin. HTLV-1-induced diseases range between inflammatory, neurodegenerative and malignant disorders. These include uveitis, dermatitis, arthritis, bronchiectasis (Reviewed in (3), and the HTLV-1 associated myelopathy/tropical spastic paraparesis (HAM/TSP), leading to a chronic neurological disease of the central nervous system (7, 8). Yet, the most aggressive form of HTLV-1-associated disorders is adult T cell leukemia (ATL) (9). ATL, discovered in Japan (9), is a hematological neoplasm with dismal prognosis. ATL develops after a very long latency period exceeding 50 years in some patients (reviewed in (10, 11). It is characterized by the clonal expansion of mature activated T cells (CD3^+^ CD4^+^ CD5^+^ CD7^-^ CD8^-^ CD25^+^) (12), and is subdivided into four clinical subtypes (acute, lymphoma, chronic, and smoldering) (13). “Indolent ATL” regroups the smoldering and chronic subtypes, while “aggressive ATL” describes the acute and lymphoma subtypes. Among all peripheral T cell lymphomas, ATL associates with the worst prognosis (14), with a 5-year OS predicted at 55, 31, 10 and 8% in the smoldering, chronic, lymphoma and acute subtypes respectively (15).

Undeniably, HTLV-1 also predisposes patients to profound immunosuppression and severe opportunistic microbial infections such as *Pneumocystis jiroveci*, *Cryptosporidium parvum*, fungal infections, activation of the Cytomegalovirus (16–18), *Strongyloides stercoralis* (19), *Staphylococcus aureus* (20), *Mycobacterium tuberculosis* (18), *Sarcoptes scabiei* (21). Moreover, higher bloodstream infections correlate with higher HTLV-1 proviral loads in patients (22).

It is intriguing how the same virus causes vastly distant diseases, and this process is highly modulated by the host/virus interplay. In that sense, host factors ostensibly play a key role in the different pathogenic outcomes of HTLV-1 infections. Not only HAM/TSP and ATL develop in different populations of HTLV-1 carriers but a flagrant immunological difference between the two categories of patients is well established. HTLV-1 carriers and HAM/TSP patients exhibit a Th-1 immune profile, while ATL patients display a Th-2/Treg response [reviewed in (23)]. It is also acknowledged that HTLV-1-specific cytotoxic T lymphocytes (CTLs) are highly activated in HAM/TSP patients, but are weaker in ATL patients, and these reduced CTLs predict a risk factor for the development of ATL (24–29). In addition, type-I interferon (IFN) plays a role in the differential suppression of HTLV-1 transcript levels between both types of patients (30), emphasizing a key role of the innate immune response, as another host determinant, in the modulation of HTLV-1 associated diseases (30). Moreover, the cytokine profile in the serum varies between HAM/TSP and ATL. Indeed, IL-10 levels are elevated in the serum of ATL patients (31), while IFN-γ, TNFα, CXCL9, and CXCL10 pro-inflammatory cytokines and chemokines are elevated in HAM/TSP patients (32).

At the viral level, the status of expression of viral proteins is critical in eliciting host immune responses, hence modulating HTLV-1 pathogenesis. Two main viral regulatory proteins, Tax and HBZ, play an essential role in this process. Recently, a dose-dependent increase in interferon (IFN)-γ and interleukin (IL)-8 was demonstrated in response to increasing doses of Tax^+^ HBZ^+^ small extracellular vesicles, and the expression of these two viral proteins in the small extracellular vesicles correlated with the proviral load and inflammatory markers in HTLV-1 carriers (33).

In this review, we will focus on ATL and on the interplay between these two viral proteins (Tax and HBZ) with the host innate immunity in modulating ATL leukemogenesis and its therapeutic responses.

## 2 HTLV-1 encoded proteins with emphasis on Tax and HBZ

The HTLV-1 provirus is flanked by the 5’ and 3’ “Long Terminal Repeat” sequences. HTLV-1 genome encodes for the characteristic structural retroviral genes (*gag, pol*, and *env)*, in addition to numerous accessory and regulatory proteins. Indeed, the pX region of the provirus has six open reading frames, five on the plus-strand and one on the minus-strand. After alternative splicing, the encoded proteins include Tax, Rex, the HTLV-1 basic leucine zipper protein (HBZ), p8/p12 (where p8 is derived from proteolytic cleavage of p12), p13, p21 and p30 (reviewed in (34, 35). It is well documented that during the long latency period, Rex regulates the post-transcriptional viral gene expression and the stability of the viral transcripts, while p12, p13 and p30 contribute to viral persistence through degradation of Major Histocompatibility Class-1 (MHC-I), alteration of T-cell receptor signaling, and suppression of Tax expression (36). More recently, the effect of monocytes and NK cells, was investigated in primary HTLV-1 infection of macaques. Exposure of animals to an HTLV-1 p12 knock-out mutant demonstrated an impaired infectivity, which was fully restored only when NK cells were depleted. Moreover, the chief role of NK cells in primary infection and the role of p8/p12 in inducing viral persistence in monocytes and in offsetting the cytotoxic effect of NK and CD8+ T cells was demonstrated (37).

Among all described regulatory proteins, Tax and HBZ proteins were lengthily studied and are tightly allied to HTLV-1 pathogenesis (38–41). While Tax is encoded by a sense mRNA, and upregulates various host genes promoting cell activation and proliferation [reviewed in (42–45)], HBZ is encoded by the minus strand of the pX region, and plays several roles, mostly counteracting Tax-induced cellular phenomena (**Table 1**) (see sections below) (35, 47, 53, 64).

**Table 1:** Summary of some antagonistic cellular effects of Tax and HBZ.

Tax	HBZ
Tax activates NF-κB, CREB, AP-1, and NF-AT (reviewed in (42, 43, 46).	HBZ suppresses CREB, AP-1, NF-AT and classical NF-κB pathways (47).
HTLV-1 Tax protein is undetectable in freshly isolated peripheral blood mononuclear cells from HTLV-1-infected individuals, but is rapidly induced in *ex-vivo* cultures (48).	HBZ mRNA is continuously detectable by RT-PCR (49, 50), and small amounts of HBZ protein were detected in primary ATL cells (51). The cytoplasmic/nuclear localization of HBZ may play a role in HTLV-1 oncogenesis (52).
High Tax levels induce senescence (39, 53–55).	HBZ expression counteracts Tax-induced senescence (39, 53).
Tax is a major target antigen for HTLV-1-specific CTLs (24, 56, 57).	Lower HBZ-specific CTLs (56).
Tax promotes IL-10 production (58, 59).	HBZ promotes IL-10 production (47, 60).
Tax promotes TGF-β production but suppresses TGF-β/Smad signaling in HTLV-1-infected cells (61, 62).	HBZ enhances TGF-β/Smad signaling, inducing FOXP3, which is frequently expressed in ATL cells (63).

### 2.1 Tax oncoprotein: A major factor in ATL leukemogenesis

#### 2.1.1 ATL-derived cells are dependent on Tax expression for their survival

Tax is a 40 kDa protein exhibiting a key role in transformation and oligoclonal expansion of virally infected cells, hence ATL initiation and progression (12, 65). Tax protein is not detectable in most ATL cells (66–68), possibly due to multiple DNA methylations identified at its 5’LTR promoter or deletions of this 5’LTR [For a review (43)]. Some studies suggested that the undetectable Tax protein levels are also due to its strong immunogenic properties, ultimately leading to the rapid elimination of Tax expressing cells by the host immune system (69–71). Despite these undetectable levels, silencing of Tax in HTLV-1-infected and ATL derived cells results in cell death, pinpointing the dependence of these cells on Tax continuous expression (58, 72). Moreover, Tax sporadic bursts occur in a very small percentage (1-3%) of ATL-derived cells at a time, to maintain and ensure the survival of the whole malignant population (73).

#### 2.1.2 Tax is oncogenic and interferes with key cellular pathways inducing leukemogenesis

Tax transactivates the plus-strand transcription by recruiting cAMP response element binding protein (CREB) and CBP/p300 and P/CAF transcriptional coactivators to Tax response elements (TREs) (74). Tax alters key cellular pathways controlling cell migration, virological synapses, and protein intracellular distribution (41–43, 74). In addition, Tax interferes with the cellular epigenetic machinery (75), down-regulates the expression of various microRNAs (76–79) and increases angiogenesis, invasion and extravasation of ATL cells, hence affecting the cellular microenvironment (80, 81).

Tax-mediated cellular consequences are partly due to its post-translational modifications (82–85), which allow its shuttling between different cellular compartments, enabling it to interfere with/activate a plethora of essential cellular regulators (41). Tax is primarily nuclear (Semmes and Jeang,1996; Bex et al.,1997), and colocalizes with various components of the NF-κB pathway (Bex et al.,1997), SUMO-1, 2, and 3 (Lamsoul et al., 2005; Nasr et al., 2006) and the SUMO-E2 ligase, Ubc-9 (Kfoury et al., 2011). Despite its abundant nuclear localization, Tax cytoplasmic expression was also described (Burton et al., 2000; Cheng et al., 2001). Indeed, Tax localizes with the microtubule organizing center, and with virological synapses (Igakura et al., 2003; Alefantis et al., 2005; Kfoury et al., 2008; Nejmeddine et al., 2009). More importantly, Tax cytoplasmic localization targets IκB-α/β for proteasomal-mediated degradation, to activate the NF-κB pathway (Nicot et al., 1998), paramount for the proliferation and survival of infected T cells (43, 84, 86–95). The activation of this pathway has pleotropic functions on top of which is the modulation of the host immune response (see section 2.1.3 below). Indeed, Tax-mediated-constitutive NF-κB activation occurs at the very early stages of HTLV-1 infection, and this pathway (canonical and non-canonical) remains constitutively activated in Tax expressing cells, ATL derived cell lines, and freshly isolated ATL cells. However, persistent Tax-induced NF-κB activation results in cellular senescence (53, 54, 96, 97), potentially offering a further explanation of the undetectable levels of Tax protein *in vivo*. Yet, recent studies suggested that the evasion from replicative senescence in HTLV-1 infected cells is achieved through reactivation of human telomerase (hTERT), and highlighted a role of Tax in the transcriptional activation of the hTERT promoter, but also in hTERT enzymatic activity, through Tax-mediated NF-κB activation (98, 99).

Tax also induces genomic instability through inhibition of cell cycle checkpoints (100–102), DNA repair mechanisms (103, 104), induction of chromosome instability (105) and aneuploidy (35, 106). Besides, Tax functionally inactivates p53 (107), and inhibits p53-induced apoptosis *via* cytoplasmic sequestration of CBP/p300 (108). Altogether, these Tax-mediated cellular phenomena result in increased proliferation and accumulation of somatic mutations due to profound genomic instability.

Finally, Tax oncogenic capacity is well recognized, as its sole expression transforms T cells *in vitro*, induces leukemia in transgenic mice (109–115) and transformation in *Drosophila* transgenic flies (116). Nevertheless, primary ATL cells display most properties of Tax expressing cells (117), and carry somatic mutations mimicking Tax cellular effects, in particular mutations targeting the T-cell receptor and the NF-κB pathways (106, 118).

#### 2.1.3 Immunological consequences of HTLV-1 infection and Tax expression

The interplay between HTLV-1 and the innate immune system was well studied (For a review (119). In the cytoplasm of infected cells, HTLV-1 viral RNA carrying 5-triphosphate is detected by the pattern recognition receptor-1 (RIG-I), culminating in the transcription of the interferon response factor-3 (IRF3) (119). This triggers the activation of the interferon anti-viral response. HTLV-1 can also infect dendritic cells (DCs), which are the foremost producers of type I interferon (120, 121). Cell–cell HTLV-1 infection induces type-I IFN production in plasmacytoid DCs (122). Furthermore, in *de novo* infection with cell-free HTLV-1, pDCs or monocytes produce type I IFN through TLR7 or STING signaling pathways, seemingly recognizing HTLV-1 RNA or its reverse transcribed intermediate DNA (123, 124). To counteract this response, HTLV-1 induces the expression of the suppressor of cytokine signaling gene SOCS1. Indeed, Tax interacts with and stabilizes SOCS1, an inhibitor of interferon signaling to inhibit RIG-I-dependent antiviral signaling and hijacking anti-viral IFN signaling (125). Another effect of Tax counterpoising type I IFN responses was also described. Indeed, Tax suppresses the TBK1 kinase which phosphorylates IRF3 impairing the production of type I IFN (126).

As previously mentioned, Tax protein levels are undetectable *in vivo* (49, 66, 68, 127). Several mechanisms were proposed to explain this finding. Tax expression triggers a strong CTL response (128, 129) and HTLV-1 infected cells and ATL cells frequently reduce the expression of Tax, to evade this CTL-mediated lysis and maintain the *in vivo* viral persistence (64, 130–134). In addition to Tax specific CTLs, anti-Tax antibodies are reported in ATL patients, pointing to the expression of the protein *in vivo*, even if at undetectable levels (135). Moreover, donor derived anti-Tax CTL were described following allogeneic hematopoietic cell transplantation for ATL (136). Prominently, the efficacy of a Tax peptide-pulsed dendritic cell vaccine in treating Tax-positive ATL patients was highlighted, further capitalizing on *in vivo* expression of Tax (137).

More recently, the role of Tax in modulating three members of the Pim serine/threonine kinases to enhance survival and inhibit apoptosis, was elucidated. Indeed, Tax increased Pim-1 and Pim-3 expression and decreased Pim-2 expression, while the three members of Pim family bind Tax, to lessen its expression in response to increased CTL responses. This feedback regulatory loop between the viral and cellular proteins suggests a potential modulation by Pim kinases of the immune escape of HTLV-1-infected cells, through partial suppression of the host immunogenic responses favoring the persistence of the virally-infected cells (138).

Finally, targeted therapies against Tax led to selective growth arrest and apoptosis *in vitro* and *in vivo*. In that sense, treatment with arsenic trioxide (AS) and interferon-alpha (IFN), which induces Tax proteasomal degradation, resulted in selective cell death of ATL cells, eradicated murine ATL through abrogating the activity of ATL leukemia initiating cells (LIC), and ensured long-lasting responses in ATL patients (See section below) (31, 58, 109, 139–142).

Finally, Tax-mediated constitutive activation of the NF-κB pathway results in a significant expression in cytokines and their receptors (43, 90, 117, 143, 144), notably Interleukin IL-6/IL6R, IL-2/IL2R, IL-9, IL-15, IL-13, interferon-γ (IFN-γ), tumor necrosis factor-beta (TNF-β), and the chemokine (C-C motif) ligand 2 (CCL2), which contribute to inhibition of apoptosis and enhanced survival of HTLV-1 infected cells (145, 146).

### 2.2 HTLV-1 basic leucine zipper (HBZ)

#### 2.2.1 HBZ attenuates Tax-mediated cellular processes

HBZ, a bZIP nuclear factor, is encoded by the minus-strand of the HTLV-1 provirus (39, 64, 147). HBZ transcription occurs at the 3’LTR promoter, generating two transcripts, the spliced sHBZ and the unspliced usHBZ transcripts (64). The expression of sHBZ is four times higher than that of unHBZ in both HTLV-1 infected and ATL cells (148). Unlike Tax, HBZ is persistently expressed *in vivo*, but at a low level (50). This might be due to the absence of DNA methylation, the intact 3’ LTR promoter, and the lack of abortive mutations in *hbz* gene. In spite of the low expression levels and the low T cell immunogenicity (149), an effective CTL response to HBZ correlates with a low proviral load *in vivo* (56, 149, 150). Furthermore, the localization of HBZ differs according to different HTLV-1 associated diseases. While HBZ is exclusively localized in the cytoplasm of HTLV-1 asymptomatic carriers and HAM/TSP patients, it exhibits a nuclear localization in ATL cell lines. In ATL patients, HBZ localizes to the cytoplasm and the nucleus of cells irrespective of the clinical status, but with a pronounced preference for the cytoplasmic localization, suggesting a role of HBZ cytoplasmic/nuclear translocation in HTLV-1 oncogenesis (52).

HBZ belongs to the basic leucine zipper protein class. As such, it controls the DNA binding or transcriptional activities of CREB-2, JunB, and c-Jun (AP-1) (134). By binding CREB-2, HBZ bZIP interacts with CREB/CREB-2, preventing it from binding to Tax-responsive element (TRE) and CRE, hence inhibiting Tax-mediated HTLV-1 transcription from the 5’LTR (64, 151). HBZ also induces T-cell proliferation through interaction with the activator protein 1 (AP1) superfamily proteins, mostly JunD (152). HBZ/JunD heterodimer enhances the transcription of the human telomerase reverse transcriptase (hTERT), which may promote cell proliferation (152). HBZ also inhibits the canonical Wnt pathway, which is deleterious for ATL development, and upregulates the transcription of Wnt5a, promoting the proliferation of ATL cells (153). Importantly, HBZ knock-down (50) or knock-out (154) impede cell proliferation (155).

At the functional level, HBZ is almost as pleiotropic as Tax (156), and many HBZ functions oppose Tax-induced cellular effects (**Table 1**). Precisely, HBZ inhibits Tax-mediated transcriptional activation of CREB, AP-1, NF-κB, and Wnt (157, 158). In addition, HBZ inhibits the canonical NF-κB pathway (157), alleviating Tax-induced cellular senescence (97). In an *in vivo Drosophila melanogaster* fly model, HBZ expression failed to activate NF-κB or to induce transformation or senescence, yet HBZ successfully activated epigenetic core components leading to consequent epigenetic changes (53). Strikingly, HBZ expression in *tax* transgenic flies prohibited Tax-induced NF-κB activation, preventing both malignant proliferation and senescence (53).

#### 2.2.2 HBZ induces inflammation and offsets anti-Tax immune response

HBZ induces the expression of CCR4 to promote cell migration and proliferation of HTLV-1-infected cells (159). As previously mentioned, Tax-expressing cells constitute a major target of CTL *in vivo* (160, 161), due to the elevated immunogenic properties of Tax. In contrast, HBZ is less immunogenic than Tax and anti-HBZ antibodies are rarely detected in infected patients (156). As such, through the continuous expression of HBZ, which offsets Tax expression (162), HTLV-1 infected cells lessen Tax expression to evade the host immune response (24, 57).

Despite its low immunogenicity, HBZ can induce inflammation. Indeed, the vast majority of *hbz*-transgenic mice develop a spontaneous systemic inflammatory disease (163). Interestingly, HBZ also stimulates the TGF-β/Smad pathway, upregulates Foxp3 expression, hence converting the T cell population into Tregs (164), to reduce the immune response (165). Likewise, HBZ promotes the secretion of IFN-ɣ in *hbz* transgenic mice, highlighting the role of HBZ in the induction of inflammation (166). Moreover, HBZ impairs cell-mediated immunity in *hbz* transgenic mice which fail to mount an optimal Th1 immune response upon challenge with *Listeria monocytogenes* or herpes simplex virus (150). In *hbz* transgenic flies, HBZ expression failed to activate NF-κB, a key pathway in the activation of the immune response (53). Indeed, HBZ attenuates the canonical NF-κB pathway, decreasing the expression of genes associated with innate immunity and inflammatory responses (157). Remarkably, HBZ totally abrogates Tax-activated canonical NF-κB, enabling cells to escape senescence and to proliferate incessantly (53, 167). HBZ also affects the transcription of several NF-κB target genes such as IL-8, IL-2RA, VEGF, CCND1, VCAM-1, and IRF4 (39, 168).

## 3 Interleukin-10 in ATL: Interplay with Tax and HBZ and role in immunosuppression

Interleukin-10 is an immunosuppressive cytokine exhibiting high levels in ATL patients and leading to an immunosuppressive profile (31, 169). IL-10 plays a role in the proliferative capacity of ATL cells through its downstream activation of STAT3 signaling (59). Recently, IL-10 was shown to be chiefly produced by the CD25^+^ cells (58), and a critical role of Tax in its production was depicted. Indeed, silencing Tax in HTLV-1 transformed or ATL derived cell lines abrogated IL-10 levels in these cells (58). Other cells and/or factors may also contribute to elevated IL-10 levels. Indeed, T helper cells, Tregs, monocytes, macrophages, and dendritic cells may produce IL-10. Moreover, the microbiome in HTLV-1 infected patients may contribute to these elevated IL-10 levels. In that sense, the predominant association of *Strongyloides stercoralis* with ATL may induce IL-10 and TGF-β (170). HBZ also modulates IL-10, through induction of expression and induced-promoter acetylation levels of TIGIT, Foxp3 and CCR4 (171). Moreover, the prolonged IFN activation by persistent viral infection can lead to an IL-10-predominant cytokine imbalance (172, 173).

## 4 Immunotherapies in the clinical management of ATL

ATL management remains intricate, after more than four decades of research. Attempts to tackle ATL by targeting leukemic cells with chemotherapy and monoclonal antibodies, without targeting HTLV-1, have failed [reviewed in (45)]. Despite slight improved outcomes with chemotherapy in newly diagnosed aggressive ATL, particularly the lymphoma subtype (174, 175), chemotherapy alone exhibits only a minimal effect on long-term survival, specifically in the acute subtype (11, 176). Allogeneic hematopoietic cell transplantation (HCT) is used in ATL (Iqbal et al., 2019), and improves the long-term survival in around one third of transplanted patients (177, 178). Yet, less that 10% of ATL patients can make it to transplant and hence the cure options using this approach do not exceed 5% of ATL patients (Hishizawa et al., 2010; Bazarbachi et al., 2014).

Since ATL is secondary to HTLV-1 infection, the combination of two antiviral agents, AZT and IFN was investigated in ATL. High response rates using this combination were achieved in newly diagnosed and relapsed ATL patients (10, 175, 176, 179–188). The smoldering and chronic subtypes benefited most from AZT/IFN which became the standard treatment of indolent ATL in most parts of the world (10, 175, 176, 180, 185, 189, 190). At the molecular level, AZT/IFN inhibits the reverse transcriptase activity and modifies the clonality pattern in responding ATL patients (191–193). Despite this clinical improvement, AZT/IFN was not curative and patients with acute and lymphoma ATL remained a population with unmet medical need.

Due to the importance of the host immune responses and the host microenvironment, in the progression of ATL, immunotherapy using monoclonal antibodies (mAb) and immune-modulatory drugs was investigated (10, 11, 194, 195). Tested mAbs mostly targeted CCR4, CD25, CD30, CD52 and the surface transferrin receptor (196–198). The humanized antibody mogamulizumab, targeting CCR4 expressed on ATL cells (197), was tested and phase I/II clinical trials proved its efficacy in patients with relapsed/refractory CCR4^+^ ATL (199). In newly diagnosed ATL patients, mogamulizumab combined with dose-intensified chemotherapy improved response rates in the peripheral blood, but failed to improve progression free survival or overall survival (200).

The efficacy of an anti-CD25 antibody, targeting CD25 highly expressed on ATL cells yielded some clinical response in indolent ATL (198). A24 mAb directed against the surface transferrin receptor induced apoptosis of ATL cell lines or primary ATL cells *in vitro* (201, 202) Alemtuzumab (Campath-1H), a chimeric humanized antibody that binds to the CD52 glycoprotein, led to promising, but short overall response rates in acute, chronic and lymphoma ATL (203). The anti-PD-1 antibody, nivolumab, was also investigated in several phase I/II clinical trials but unfortunately led to a rapid progression of ATL (204). Finally, the immunomodulatory drug, lenalidomide exhibited a significant anti-leukemic activity, in relapsed/recurrent ATL (205). Recently, low dose lenalidomide was proposed as a maintenance therapy of ATL, and resulted in continuous complete remission in a patient with acute ATL lasting more than 24 months (195). Finally, the anti-CD30 monoclonal antibody brentuximab vedotin (BV), used in several clinical trials including patients with relapsed/refractory CD30^+^ ATL patients, yielded promising results (196, 206).

## 5 Dual targeting of the innate immune response and viral oncoproteins: An innovative therapeutic approach for the treatment of ATL

The key role of Tax and HBZ in ATL development and maintenance of the leukemic phenotype highlights the potential importance of ATL therapeutic approaches directly targeting these viral proteins or indirectly targeting their downstream cellular targets or inducing antiviral immunity. In that sense, the combination of arsenic trioxide (AS) and interferon-α (IFN) selectively induced cell cycle arrest and apoptosis of ATL cells *in vitro* (139). This was associated with a reversal of the constitutive activation of NF-κB and delayed shut down of cell cycle-regulated genes secondary to proteoasomal-mediated Tax degradation (207–209). *In vivo*, AS/IFN cured Tax-driven murine ATL through leukemia initiating cell (LIC) eradication (109). AS/IFN-induced abolition of ATL LIC activity required IL-10 expression shutoff. Indeed, loss of IL-10 secretion by ATL cells, triggered the production of inflammatory cytokines by the innate immune microenvironment, namely NK cells and macrophages, hence mediating the clearance of ATL cells. Strikingly, anti-IL-10 monoclonal antibodies significantly increased the efficiency of AS/IFN therapy (58), and treatment of murine ATL with the triple combination of AS/IFN/anti-IL-10 monoclonal antibody cured 80% of mice and significantly decreased LIC activity in serial transplantation assays (58). Overall, these results highlight the potential dual targeting of malignant ATL cells and their immune microenvironment and provide a strong rational to test the therapeutic effect of this triple combination in ATL patients.

The importance of such a dual targeting of viral oncoproteins and the immune microenvironment, was further strengthened by vaccination approaches against Tax, HBZ or both. A Tax peptide-pulsed dendritic cell (DC) vaccine, designed to augment Tax-specific CTL response, led to favorable clinical outcomes in a pilot clinical trial (137), and two patients survived for more than 4 years after vaccination (136). A recombinant vaccinia virus (rVV) that induced an HBZ-specific T-cell response, improved the survival of HBZ-induced lymphoma-challenged mice (210). And finally, THV02, comprising two lentiviral vectors encoding for a peptide deriving from the viral proteins Tax, HBZ, p12I and p30II, and to be used in a prime/boost regimen, induced a promising cellular response in animal models (Hermine et al. personal communication).

## 6 Conclusions

**Figure 1:**
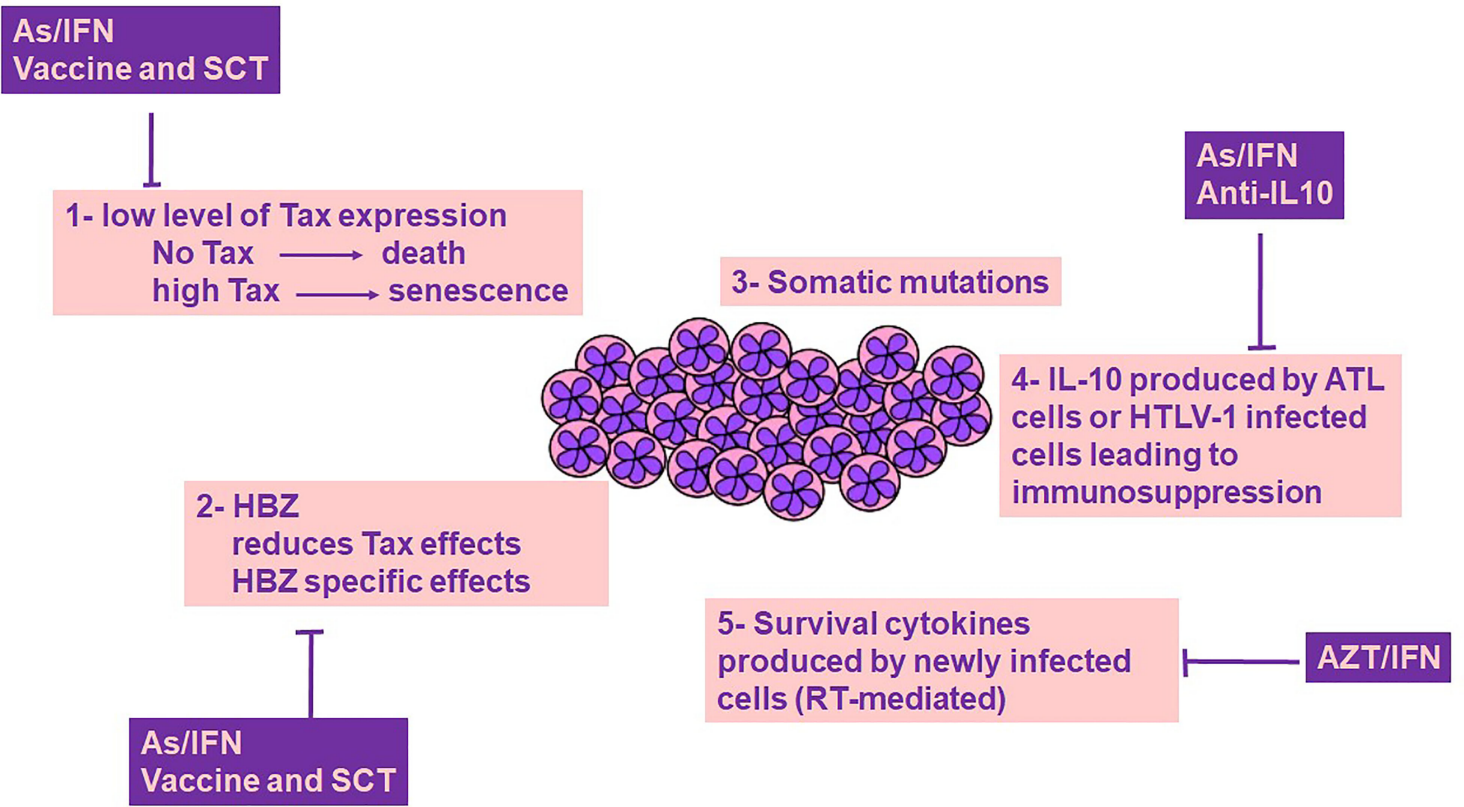
ATL cells survival: a cross-talk between genetics, viral proteins and immune-microenvironment. Survival of ATL cells requires Tax expression, yet Tax is highly immunogenic, and its expression at high levels drives senescence, a cellular fate counterbalanced by HBZ. Tax induced genetic instability results in the accumulation of somatic mutations. Both Tax and HBZ promote IL-10 expression, a key cytokine contributing to ATL cell survival and host immunosuppression. Newly infected T cells produce cytokines that contribute to the survival of ATL cells. The role of Tax/HBZ and IL-10 in ATL leukemogenesis highlights the importance of dual targeted therapies including anti-viral therapies and targeted therapies against viral oncoproteins and IL-10, as a promising curative avenue for ATL. AZT/IFN, Zidovudine and Interferon-alpha; As/IFN, Arsenic trioxide and Interferon-alpha; SCT, Stem Cell Transplantation; IL-10, Interleukin-10; RT, Reverse transcriptase.

ATL is a virally-driven malignancy that associates with dismal prognosis. The clinical management of ATL remains difficult, partially due to the incomplete understanding of the intimate relationship between HTLV-1 and its induced forefront immune response. Indeed, the intricacy of disease mechanisms following HTLV-1 infection is a consequence of the interplay between the host immune responses in concert with HTLV-1 proteins including Tax and HBZ. Despite the intensive literature on the plethora of functions of these viral oncoproteins, Tax and HBZ fail to induce and sustain proliferation of malignant ATL cells without IL-10 (**Figure 1**). Both Tax and HBZ upregulate IL-10 production, inducing proliferation of HTLV-1-infected cells. This effect, along with the anti-inflammatory and immunosuppressive properties of IL-10 may play a key role in switching HTLV-1 induced inflammation towards ATL. The tremendously low but not silent levels of Tax protein expression in ATL patients and the efficacy of Tax-targeted therapeutic vaccine in ATL patients highlight the impact of Tax-specific CTLs on immune surveillance of HTLV-1 infected and ATL cells. Moreover, targeted therapies leading to Tax degradation proved selective and potent efficacy against ATL cells *in vitro* and *in vivo*. Murine preclinical models of ATL highpoint the importance of the dual targeting of the innate immune microenvironment and the viral oncoproteins. Adding pieces to the intriguing puzzle of host immunity/HTLV-1 infection is required, and future studies should include therapies that target the main driver of ATL, the HTLV-1 virus (**Figure 1**). These therapeutic options may target the viral proteins, their downstream cellular targets, along with the host immune microenvironment including HTLV-1 infected non-malignant cells.

## Author contributions

HEH and AB equally contributed in writing this review. All authors read and approved the final manuscript.

## Conflict of interest

The authors declare this review was written in the absence of any commercial or financial relationships that could be construed as a potential conflict of interest.

## Publisher’s note

All claims expressed in this article are solely those of the authors and do not necessarily represent those of their affiliated organizations, or those of the publisher, the editors and the reviewers. Any product that may be evaluated in this article, or claim that may be made by its manufacturer, is not guaranteed or endorsed by the publisher.
